# Evaluation of an inflammation-based prognostic score in patients with inoperable gastro-oesophageal cancer

**DOI:** 10.1038/sj.bjc.6602998

**Published:** 2006-02-14

**Authors:** A B C Crumley, D C McMillan, M McKernan, A C McDonald, R C Stuart

**Affiliations:** 1 University Department of Surgery, Royal Infirmary, Glasgow G31 2ER, UK; 2 Beatson Oncology Centre, Western Infirmary, Glasgow G11 6NT, UK

**Keywords:** gastro-oesophageal cancer, TNM stage C-reactive protein, albumin, survival

## Abstract

There is increasing evidence that the presence of an ongoing systemic inflammatory response is associated with poor outcome in patients with advanced cancer. The aim of the present study was to examine whether an inflammation-based prognostic score (Glasgow Prognostic score, GPS) was associated with survival, in patients with inoperable gastro-oesophageal cancer. Patients diagnosed with inoperable gastro-oesophageal carcinoma and who had measurement of albumin and C-reactive protein concentrations, at the time of diagnosis, were studied (*n*=258). Clinical information was obtained from a gastro-oesophageal cancer database and analysis of the case notes. Patients with both an elevated C-reactive protein (>10 mg l^−1^) and hypoalbuminaemia (<35 g l^−1^) were allocated a GPS score of 2. Patients in whom only one of these biochemical abnormalities was present were allocated a GPS score of 1, and patients with a normal C-reactive protein and albumin were allocated a score of 0. On multivariate survival analysis, age (hazard ratio (HR) 1.22, 95% CI 1.02–1.46, *P*<0.05), stage (HR 1.55, 95% CI 1.30–1.83, *P*<0.001), the GPS (HR 1.51, 95% CI 1.22–1.86, *P*<0.001) and treatment (HR 2.53, 95% CI 1.80–3.56, *P*<0.001) were significant independent predictors of cancer survival. A 12-month cancer-specific survival in patients with stage I/II disease receiving active treatment was 67 and 60% for a GPS of 0 and 1, respectively. For stage III/IV disease, 12 months cancer-specific survival was 57, 25 and 12% for a GPS of 0, 1 and 2, respectively. In the present study, the GPS predicted cancer-specific survival, independent of stage and treatment received, in patients with inoperable gastro-oesophageal cancer. Moreover, the GPS may be used in combination with conventional staging techniques to improve the prediction of survival in patients with inoperable gastro-oesophageal cancer.

Gastro-oesophageal cancer is the third commonest cause of cancer death in the UK. Each year, there are approximately 16 500 new cases and over 13 000 deaths attributable to the disease. Overall survival is poor with the majority of patients presenting with advanced, inoperable disease and less than 15% surviving 5 years (Cancerstats, 2004; www.cancerresearchuk.org). Despite an often short median and poor overall survival, there is marked heterogeneity in the duration of survival among patients. Therefore, there is continuing interest in prognostic factors to permit more accurate patient stratification and which will improve clinical decision making, and possibly contribute to more rational study design and analysis (Allgayer *et al*, 1997).

A small proportion of patients with inoperable, but localised oesophageal cancers may be suitable for potentially curative nonsurgical treatment with (chemo) radiation therapy; however, most frequently, these modalities are used in palliation. While such palliative treatment may confer a small survival advantage over best supportive care, it is primarily directed towards symptom relief (Middleton and Cunningham, 1995; Pyrhonen *et al*, 1995). This however may sometimes be at the expense of toxicity (Ross *et al*, 2002; Harvey *et al*, 2004) and therefore the appropriate selection of patients, most likely to benefit is of considerable importance.

Previous studies have indicated that weight loss or performance status may be associated with treatment outcome and survival in inoperable oesophago-gastric cancer (Andreyev *et al*, 1998; Chau *et al*, 2004). However, the use of weight loss as a prognostic factor remains problematical since it is often not well defined and subject to bias (Morgan *et al*, 1980; Rowland, 1990). Furthermore, performance status is recognised to be subjective (Ando *et al*, 2001).

There is increasing evidence that the presence of a systemic inflammatory response, as evidenced by elevated concentrations of C-reactive protein, is a prognostic factor independent of stage, performance status and weight loss in patients with advanced cancer (O’Gorman *et al*, 2000; Scott *et al*, 2002; Maltoni *et al*, 2005). Recently, we have shown that an elevated C-reactive protein and hypoalbuminaemia (using standardised assays and accepted thresholds for C-reactive protein and albumin concentrations) may be combined to form a score, the Glasgow Prognostic score (GPS), which has prognostic value, independent of stage and performance status, in patients with inoperable non-small-cell lung cancer (Forrest *et al*, 2003, 2004).

The aim of the present study was to assess the relationship between the GPS and survival in patients with inoperable gastro-oesophageal cancer.

## MATERIALS AND METHODS

### Patients

Patients diagnosed with inoperable gastro-oesophageal carcinoma, attending the upper GI surgical unit in the Royal Infirmary, Glasgow between the 1 January 2000 and the 31 December 2004 and who had a pretreatment measurement of C-reactive protein and albumin were studied. Patients were staged using a combination of endoscopy, CT scan of chest and abdomen, laparoscopy and endoscopic ultrasound, in addition to clinical assessment. The specific use of these modalities was dependent upon the clinical tumour features and where appropriate, assessment of fitness, cardiac and lung function testing was also performed.

The extent of tumour spread was recorded using the TNM stage. Tumours around the gastro-oesophageal junction were further classified according to site, using the Siewert system; type 1 and 2 lesions of the gastro-oesophageal junction were designated as cancers of the oesophagus. Type 3 tumours of the cardia were designated as gastric cancers.

Patients identified as being suitable for resection or radical, nonsurgical treatment given with curative intent were excluded from this analysis, as were patients who had any form of chronic inflammatory disease (e.g. vasculitis, connective tissue disorders, rheumatological conditions) and those with cancers arising in other organs. Therefore, the study group comprised patients unsuitable for either surgical resection or radical, nonsurgical treatment.

Patients who underwent palliative chemotherapy, palliative radiotherapy or endoscopic laser were considered to have had ‘active’ treatment. Patients receiving palliative care (symptom control) were considered to have had ‘supportive’ treatment. The ‘active’ treatment group was further subdivided into: chemotherapy based (chemotherapy±radiotherapy±endoscopic treatment), radiotherapy based (radiotherapy±endoscopic treatment) and endoscopic laser (laser±stent).

The study was approved by the Research Ethics Committee of Glasgow Royal Infirmary.

### Methods

Routine laboratory measurements of C-reactive protein and albumin at the time of diagnosis were carried out. The limit of detection of the C-reactive protein assay was <6 mg l^−1^. The coefficients of variation of these methods, over the range of measurements, was less than 5% as established by routine quality control.

The GPS was constructed as previously described (Forrest *et al*, 2003, 2004). Briefly, patients with both an elevated C-reactive protein (>10 mg l^−1^) and hypoalbuminaemia (<35 g l^−1^) were allocated a score of 2. Patients in whom only one of these biochemical abnormalities was present were allocated a score of 1. Patients in whom neither of these abnormalities was present were allocated a score of 0.

### Statistics

Data are presented as median and 95% CI. Grouping of the variables was carried out using standard thresholds. Univariate survival analysis was performed using the Kaplan–Meier method with the logrank test. Multivariate survival analysis and calculation of hazard ratios (HR) were performed using a Cox regression model including all covariates that were significant on univariate analysis. Deaths up to 30 June 2005 were included in the analysis. Analysis was performed using SPSS software (SPSS Inc., Chicago, IL, USA).

## RESULTS

The characteristics and survival analysis of patients with inoperable gastro-oesophageal cancer (*n*=258) are shown in Table 1. The majority were male, over the age of 65 years, had stage IV disease and had tumours of the oesophagus. Patients with stage I and II disease were not considered suitable for surgery or radical chemoradiation due to comorbidity. The majority of patients had an abnormal GPS. Of the 52 patients with hypoalbuminaemia, 45 (86%) had an elevated C-reactive protein concentration. In total, 195 patients (76%) received active treatment and the remainder received supportive care only.

**Figure 1:**
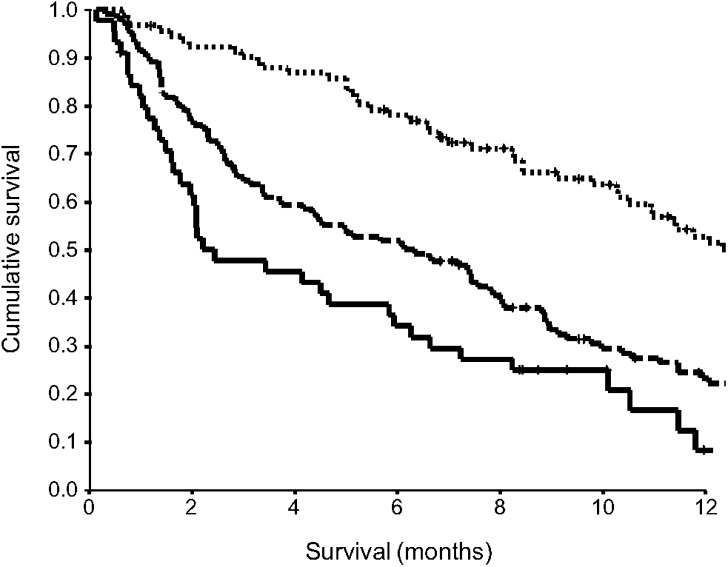
The relationship between an inflammation-based prognostic score (GPS, 0, 1, 2 from top to bottom) and survival in patients with inoperable gastro-oesophageal cancer.

The minimum follow-up was 6 months or until date of death; the median follow-up of the survivors was 12 months. During this period, 211 (82%) patients died: 202 patients of their cancer and nine of intercurrent disease. On univariate analysis, tumour site (*P*<0.05), stage (*P*<0.001), alkaline phosphatase (*P*<0.05), the GPS (*P*<0.001, Figure 1) and treatment (*P*<0.001) were significant predictors of cancer-specific survival. On multivariate analysis, age (*P*<0.05), stage (*P*<0.001), the GPS (*P*<0.001) and treatment (*P*<0.001) were significant independent predictors of cancer-specific survival (Table 2).

**Figure 2:**
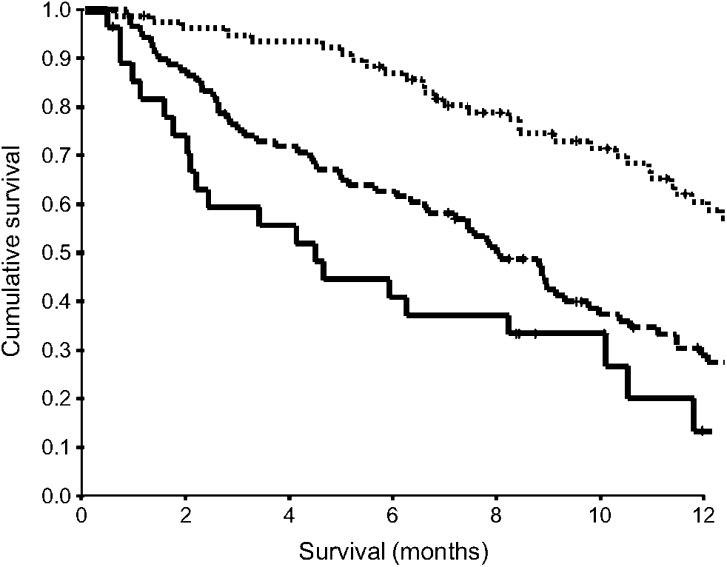
The relationship between an inflammation-based prognostic score (GPS, 0, 1, 2 from top to bottom) and survival in patients with inoperable gastro-oesophageal cancer receiving active treatment.

The characteristics and survival analysis of those patients receiving active treatment (*n*=195) are shown in Table 3. On multivariate analysis, stage (*P*<0.001), the GPS (*P*<0.001, Figure 2) and treatment (*P*<0.01) were significant independent predictors of cancer-specific survival.

The characteristics and survival analysis of those patients receiving supportive treatment (*n*=63) are shown in Table 4. On multivariate analysis, only stage (*P*<0.05) was a significant independent predictor of cancer-specific survival.

The relationship between stage, the GPS and the 12-month survival rate in those patients receiving active treatment is shown in Table 5. The 12-month cancer-specific survival in patients with stage I/II disease receiving active treatment was 67 and 60% for a GPS of 0 and 1, respectively. For stage III/IV disease, the 12-month cancer-specific survival was 57, 25 and 12% for a GPS of 0, 1 and 2, respectively.

## DISCUSSION

In the present study the presence of a systemic inflammatory response, reflected in the GPS, predicts cancer-specific survival, independent of tumour stage, in patients with inoperable gastro-oesophageal cancer. Moreover, we have shown how the GPS might be used in combination with stage to improve the prediction of survival. It may be that this simply derived inflammation-based score will be a useful tool in the prediction of survival and possible stratification, at diagnosis, of patients with inoperable gastro-oesophageal cancer.

It was of interest that, in the present study, only seven (14%) patients had hypoalbuminaemia in the absence of an elevated C-reactive protein concentration. This is consistent with the concept that the development of hypoalbuminaemia is often secondary to an ongoing systemic inflammatory response (McMillan *et al*, 2001; Al Shaiba *et al*, 2004). Glasgow Prognostic score may thus reflect both the presence of an ongoing systemic inflammatory response (C-reactive protein) and the progressive nutritional decline (albumin) of the patient with advanced cancer.

The mechanism by which a systemic inflammatory response might influence cancer survival in these patients is not clear. However, it may be that the presence of a systemic inflammatory response and the associated nutritional decline (McMillan *et al*, 1998; Scott *et al*, 2002) influences tolerance and compliance with active treatment (Bromwich *et al*, 2004; Forrest *et al*, 2004). Indeed, Andreyev *et al* (1998) reported that the poorer outcome of chemotherapy in advanced gastrointestinal cancer patients with weight loss appeared to be as a result of receiving less chemotherapy, due to toxicity, rather than poorer tumour response.

When the relationship between the GPS and 12-month survival rate was examined in patients with stage III/IV disease receiving active treatment, there was approximately a five-fold decrease in the survival rate between those patients with a GPS of 0 (57%) and those with a GPS of 2 (12%). This suggests that there is a subgroup of patients who derive little survival benefit from active treatment. However, it is important to remember that treatment in these patients is given with palliative intent and survival data do not reflect end points of palliation. This aspect is being explored further in ongoing work, however the identification of a patient subgroup with limited prognosis, through the use of a simple reliable prognostic score, may aid the treatment decision-making process. In particular, it would seem inappropriate to subject such patients to potentially toxic treatments if simpler palliative options exist.

In summary, the prognosis for patients diagnosed with inoperable gastro-oesophageal cancer, even with active treatment, remains poor. The presence of a systemic inflammatory response (an elevated GPS) appears to be a useful indicator of outcome among these patients, independent of stage. Moreover, the GPS has the advantage of being simple to measure, routinely available and well standardised.

## Figures and Tables

**Table 1:** Clinical characteristics and cancer-specific survival in patients with inoperable gastro-oesophageal cancer: univariate survival analysis

	**Patients 258 (100%)**	**Survival (months) median (95% CI)**	***P*-value**
*Age (years)*			
<65	91 (35)	8.0 (7.0–9.0)	
65–74	64 (25)	6.6 (2.5–10.8)	
>75	103 (40)	7.4 (4.8–10.1)	0.664
			
*Sex*			
Male	166 (64)	7.4 (5.7–9.1)	
Female	92 (36)	8.0 (6.2–9.9)	0.728
			
*Tumour type*			
Adenocarcinoma	187 (73)	8.2 (6.2–9.6)	
Squamous	71 (27)	6.6 (4.9–8.3)	0.979
			
*Tumour site*			
Oesophagus	142 (55)	8.9 (6.8–11.1)	
Gastric	116 (45)	6.6 (4.0–9.3)	0.042
			
*TNM stage*			
I	29 (11)	20.5 (13.3–27.7)	
II	27 (11)	11.8 (8.3–15.3)	
III	64 (25)	9.8 (8.1–11.5)	
IV	138 (53)	4.5 (2.4–6.5)	<0.001
			
*Alkaline phosphatase (U l*^*−1*^)			
Tertile 1 (*n*=85)	145 (19–176)^a^	8.4 (6.7–10.2)	
Tertile 2 (*n*=85)	199 (176–233)	8.9 (6.8–11.1)	
Tertile 3 (*n*=84)	325 (235–2396)	5.0 (2.1–7.9)	0.050
			
*GPS*			
0	92 (36)	13.6 (9.2–18.1)	
1	121 (47)	6.3 (4.2–8.5)	
2	45 (17)	2.4 (0.5–4.4)	<0.001
			
*Treatment*			
Active	195 (76)	10.1 (8.6–11.6)	
Supportive	63 (24)	2.1 (1.3–2.8)	<0.001

a Median (range).

GPS=Glasgow Prognostic score.

**Table 2:** Clinical characteristics and cancer-specific survival in patients with inoperable gastro-oesophageal cancer: multivariate survival analysis

	**Patients (*n*=258)**	**Hazard ratio (95% CI)**	***P*-value**
Age (<65/65–74/>75 years)	91/64/103	1.22 (1.02–1.46)	0.032
Sex (male/female)	166/92	1.07 (0.80–1.45)	0.642
Tumour type (adenocarcinoma/squamous)	187/71	1.28 (0.87–1.89)	0.210
Tumour site (oesophagus/gastric)	142/116	1.36 (0.96–1.92)	0.087
TNM stage (I/II/III/IV)	29/27/64/138	1.55 (1.30–1.83)	<0.001
GPS (0/1/2)	92/121/45	1.51 (1.22–1.86)	<0.001
Alkaline phosphatase (U l^−1^) (Tertiles 1/2/3)	85/85/84	1.10 (0.92–1.32)	0.300
Treatment (active/supportive)	195/63	2.53 (1.80–3.56)	<0.001

GPS=Glasgow Prognostic score.

**Table 3:** Clinical characteristics and cancer-specific survival in patients with inoperable gastro-oesophageal cancer receiving active treatment: multivariate survival analysis

	**Patients (*n*=195)**	**Hazard ratio (95% CI)**	***P*-value**
Age (<65/65–74/>75 years)	74/49/72	1.13 (0.88–1.45)	0.354
Sex (male/female)	129/66	1.14 (0.80–1.65)	0.465
Tumour type (adenocarcinoma/squamous)	142/53	1.39 (0.89–2.17)	0.154
Tumour site (oesophagus/gastric)	114/81	1.27 (0.85–1.89)	0.249
TNM stage (I/II/III/IV)	27/20/51/97	1.66 (1.36–2.03)	<0.001
GPS (0/1/2)	78/89/28	1.75 (1.35–2.26)	<0.001
Alkaline phosphatase (U/l) (Tertiles 1/2/3)	67/64/60	0.97 (0.78–1.21)	0.788
Treatment (chemotherapy/radiotherapy/endoscopic)	102/33/60	1.48 (1.15–1.90)	0.003

GPS=Glasgow Prognostic score.

**Table 4:** Clinical characteristics and cancer-specific survival in patients with inoperable gastro-oesophageal cancer receiving supportive treatment: multivariate survival analysis

	**Patients (*n*=63)**	**Hazard ratio (95% CI)**	***P*-value**
Age (<65/65–74/>75 years)	17/15/31	0.87 (0.61–1.25)	0.449
Sex (male/female)	37/26	1.17 (0.67–2.05)	0.579
Tumour type (adenocarcinoma/squamous)	45/18	0.89 (0.39–2.04)	0.785
Tumour site (oesophagus/gastric)	28/35	0.77 (0.36–1.63)	0.492
TNM stage (I/II/III/IV)	2/7/13/41	1.68 (1.08–2.64)	0.023
GPS (0/1/2)	14/32/17	1.04 (0.72–1.51)	0.824
Alkaline phosphatase (U l^−1^) (Tertiles 1/2/3)	18/21/24	1.36 (0.94–1.98)	0.105

GPS=Glasgow Prognostic score.

**Table 5:** The relationship between stage, the GPS and the 12-month cancer-specific survival rate in patients with inoperable gastro-oesophageal cancer receiving active treatment (*n*=195)

	**Stage I+II**	**Stage III+IV**	**Stage I–IV**
GPS 0	67% (*n*=31)	57% (*n*=47)	61% (*n*=78)
GPS 1	60% (*n*=10)	25% (*n*=79)	29% (*n*=89)
GPS 2	0% (*n*=6)	12 % (*n*=22)	16% (*n*=28)
GPS 0–2	62% (*n*=47)	33% (*n*=148)	40% (*n*=195)

GPS=Glasgow Prognostic score.
